# Orchestrating vesicular and nonvesicular membrane dynamics by intrinsically disordered proteins

**DOI:** 10.15252/embr.202357758

**Published:** 2023-09-08

**Authors:** Stephan J Sigrist, Volker Haucke

**Affiliations:** ^1^ Department of Biology, Chemistry, Pharmacy Freie Universität Berlin Berlin Germany; ^2^ Department of Molecular Pharmacology and Cell Biology Leibniz Forschungsinstitut für Molekulare Pharmakologie (FMP) Berlin Germany

**Keywords:** intrinsically disordered proteins, membrane contact sites, neurotransmission, synapse, vesicular and nonvesicular transport, Membranes & Trafficking, Neuroscience, Molecular Biology of Disease

## Abstract

Compartmentalization by membranes is a common feature of eukaryotic cells and serves to spatiotemporally confine biochemical reactions to control physiology. Membrane‐bound organelles such as the endoplasmic reticulum (ER), the Golgi complex, endosomes and lysosomes, and the plasma membrane, continuously exchange material via vesicular carriers. In addition to vesicular trafficking entailing budding, fission, and fusion processes, organelles can form membrane contact sites (MCSs) that enable the nonvesicular exchange of lipids, ions, and metabolites, or the secretion of neurotransmitters via subsequent membrane fusion. Recent data suggest that biomolecule and information transfer via vesicular carriers and via MCSs share common organizational principles and are often mediated by proteins with intrinsically disordered regions (IDRs). Intrinsically disordered proteins (IDPs) can assemble via low‐affinity, multivalent interactions to facilitate membrane tethering, deformation, fission, or fusion. Here, we review our current understanding of how IDPs drive the formation of multivalent protein assemblies and protein condensates to orchestrate vesicular and nonvesicular transport with a special focus on presynaptic neurotransmission. We further discuss how dysfunction of IDPs causes disease and outline perspectives for future research.

## Graphical abstract

Information transfer via vesicles or membrane contact sites is often mediated by proteins with IDRs. This review discusses how intrinsically disordered proteins drive the formation of multivalent protein assemblies and protein condensates to orchestrate vesicular and nonvesicular transport and how IDP dysfunction causes disease.

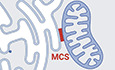

## Introduction

**Figure 1:**
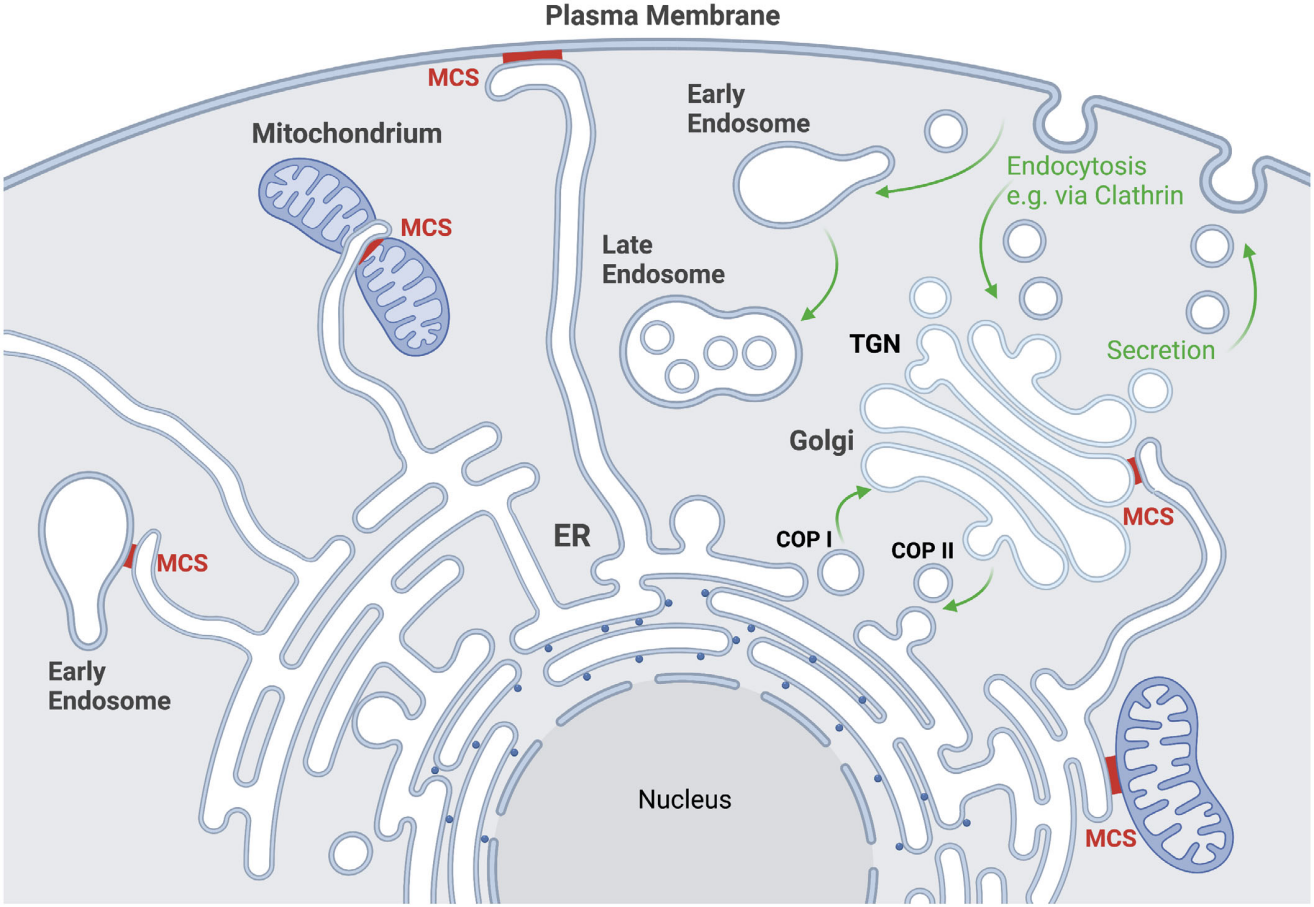
Cellular modes of material exchange: Vesicle trafficking and nonvesicular transport via membrane contact site (MCS) formation Shown are cellular locales using vesicular traffic and MCS formation for the exchange of metabolites, lipids ions, or proteins. ER, endoplasmic reticulum; COPI, coat protein complex I; COPII, coat protein complex II; TGN, trans‐Golgi network. Created with BioRender.com.

A hallmark of eukaryotic cells is the elaborate membrane system that serves to create physically and functionally distinct organelles, which enable cells to compartmentalize metabolic networks and signaling cascades. Membrane‐bound cell organelles such as the endoplasmic reticulum (ER), Golgi complex, endosomes and lysosomes, and the plasma membrane (PM) continuously exchange biomolecules (e.g., proteins, lipids, and metabolites) via vesicular and tubular carriers (hereafter referred to as vesicular transport) in processes such as the internalization of membranes (endocytosis via membrane fission) or the secretion of proteins, hormones, or neurotransmitters (exocytosis via membrane fusion; Bonifacino & Glick, 2004; Sudhof & Rothman, 2009; Bethune & Wieland, 2018; Yarwood *et al*, 2020). Vesicular transport (Fig 1) via membrane fusion, fission, and maturation processes underlies a broad range of cellular functions beyond exo‐ and endocytosis, for example, in cell morphogenesis, migration, cell signaling, or tissue formation. Organelles such as the ER and the PM also display further subcompartmental organization and heterogeneity at the nanoscale level that allow for the segregation of functional biochemical units for information processing (e.g., neurotransmitter receptors localized within the postsynaptic density; Sudhof & Rothman, 2009) and/or the vectorial transfer of biomolecules (e.g., the polarized secretion of enzymes or hormones; Bonifacino, 2014). Recent data show that changing environmental conditions or physiological stimuli such as starvation, stress, or patterns of neuronal activity impinge on vesicular transport to control cell and organismic physiology.

In addition to vesicular transport, many organelles can form stable or transient membrane contact sites (MCS) with each other (Cohen *et al*, 2018; Wu *et al*, 2018; Prinz *et al*, 2020; Fig 1). In this way, opposing organelles are tethered to each other to enable the nonvesicular exchange of lipids (e.g., cholesterol and phospholipids), ions (e.g., store‐operated calcium entry via STIM1‐ORAI channels at ER‐PM contacts), and metabolites, or to secrete neurotransmitters via a subsequent fusion event (e.g., exocytosis of synaptic vesicles (SVs) docked at the PM within the so‐called active zone (AZ; Sudhof & Rothman, 2009; Haucke *et al*, 2011). As in vesicular trafficking, MCS are subject to dynamic regulation, for example, via cell signaling cascades (e.g., involving calcium or small GTPases such as Rabs) and in response to nutrient status and metabolism (Cohen *et al*, 2018; Wu *et al*, 2018; Prinz *et al*, 2020), thereby providing a means of interorganellar communication that is independent of the formation of vesicular carriers, that is, membrane fission and fusion.

**Figure 2:**
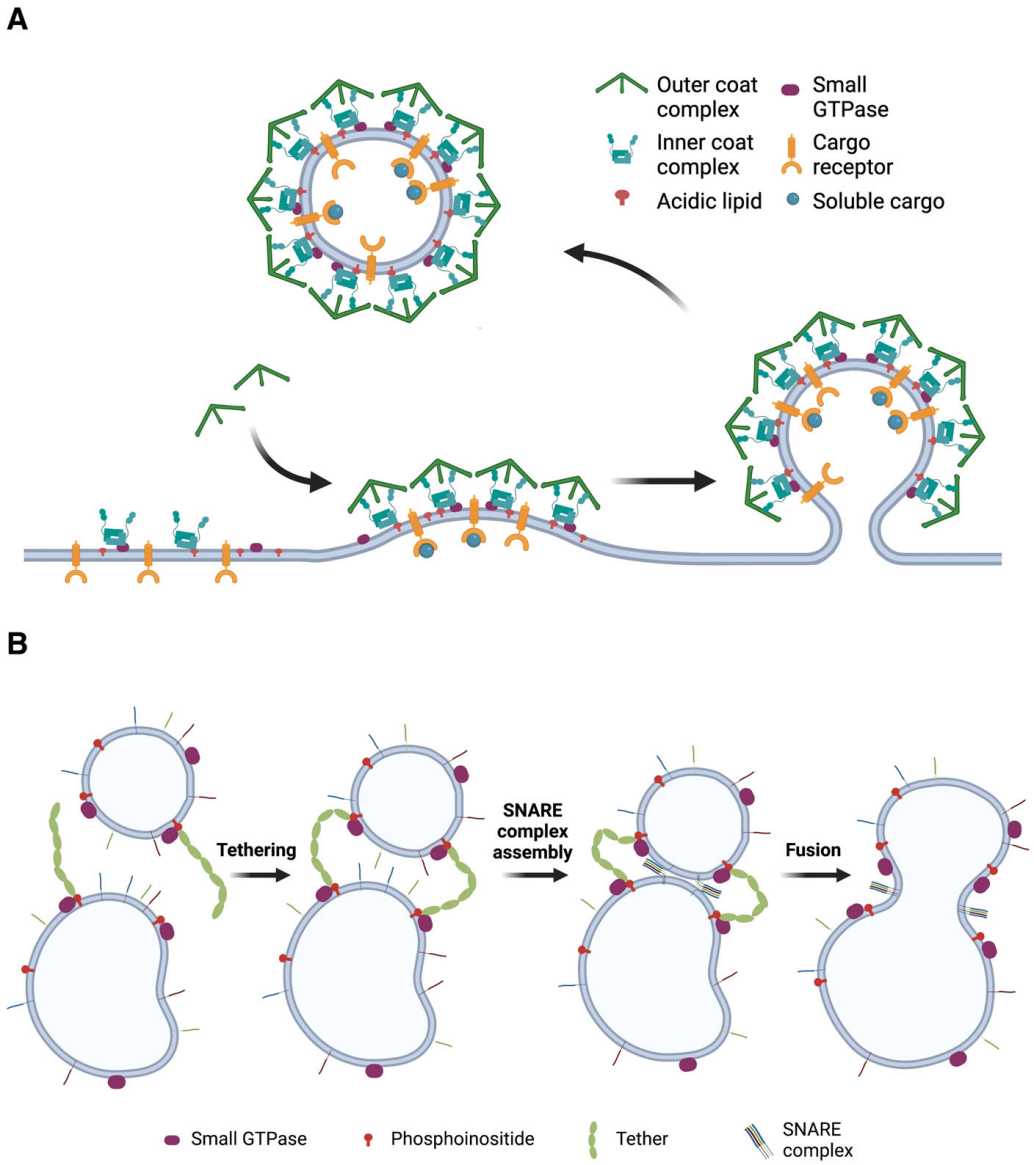
Principles of vesicular transport (A) Schematic representation of vesicle formation and budding. Vesicle coat complexes are recruited in a stepwise manner via coincident detection of cargo or cargo receptors and a specific small GTPase and/or acidic membrane lipids. (B) Schematic representation of the steps toward homotypic fusion orchestrated by tethering complexes, cognate SNARE complexes, as well as GTP‐bound Rab proteins and phosphoinositides serving as membrane identity markers. Created with BioRender.com.

Emerging evidence suggests that biomolecule and information transfer via vesicular transport and nonvesicular transport at MCS (Fig 1) share organizational principles. Membrane fusion/fission and MCS formation both require the finely tuned regulation of the interaction of proteins with membranes that capitalizes on coincident recognition of small GTPases, for example, Rab GTPases, and phosphoinositide signaling lipids (phosphorylated derivatives of phosphatidylinositol collectively referred to as PIs). PIs and Rabs act as determinants of compartmental membrane identity (Behnia & Munro, 2005) and spatiotemporal cues to direct both vesicular transport and MCS formation or dissolution, in addition to other cellular functions (e.g., in signaling; Di Paolo & De Camilli, 2006; Schink *et al*, 2016; Posor *et al*, 2022). For instance, Rab5 and phosphatidylinositol 3‐phosphate [PI(3)P] serve as spatiotemporal landmarks for endosomes and direct vesicular transport in the early endocytic pathway by facilitating both membrane fusion (Fig 2A) and formation of MCS with the ER (Simonsen *et al*, 1998; Zerial & McBride, 2001; Ohya *et al*, 2009). These MCS control endosome position and dynamics, lipid exchange, and, conversely, ER shape (Jang *et al*, 2022). Many of the membrane‐associated effector proteins recruited by co‐incident recognition of small GTPases and PIs that orchestrate vesicular and nonvesicular transport harbor extended intrinsically disordered regions (IDRs) that are essential for their functionality (Uversky *et al*, 2008; Wright & Dyson, 2015; Jamecna & Antonny, 2021; Coskuner‐Weber *et al*, 2022; Schiano Lomoriello *et al*, 2022).

In this review, we discuss how the formation of multivalent protein assemblies and phase‐separated biomolecular condensates may enable intrinscially disordered proteins (IDPs) to orchestrate vesicular and nonvesicular transport in health and disease with special emphasis on presynaptic neurotransmission.

## Vesicular and nonvesicular transport

### Vesicular transport

In vesicular transport, vesicle coat proteins are recruited to and assembled on defined nanodomains of an organelle (e.g., endocytic sites on the PM, ER exit sites) to mediate the transport of various cargos (e.g., proteins and lipids) for the biogenesis of organelles, secretion of proteins, peptide hormones or neurotransmitters, and for cargo uptake (e.g., endocytosis) into cells as well as to control plasma membrane composition (Bonifacino & Glick, 2004; Faini *et al*, 2013; Bethune & Wieland, 2018). Vesicle coat proteins serve to induce and stabilize membrane curvature and couple this to the sorting of transmembrane and luminal cargos into the emerging transport vesicle. The best studied examples of vesicle coats are the various types of clathrin‐coated vesicles defined by distinct clathrin adaptor complexes (e.g., AP‐1 to AP‐5) and coat protein I (COPI) and coat protein II (COPII) vesicles that mediate bidirectional transport at the ER–Golgi interface (Faini *et al*, 2013; Bethune & Wieland, 2018). Architecturally, the components of these vesicle coats are characterized by the presence of extended α‐solenoid and β‐propeller domains that are also found in some membrane tethers (see below) and in nucleoporin proteins that delineate the nuclear pore (Rout & Field, 2017).

Recruitment and assembly of vesicle coats typically requires coincident detection of membrane lipid composition (e.g., acidic lipids) together with proteinaceous factors, most often—though not always—the activated GTP‐bound form of a small GTPase (e.g., ADP ribosylation factor 1, Arf1; Fig 2B). In many cases, coat recruitment and polymerization are accompanied by conformational changes within vesicle coat components (e.g., clathrin adaptors) that facilitate membrane binding and couple coat assembly to the sorting of transmembrane cargo (Faini *et al*, 2013; Fig 2A). A prime example is the conformational activation of the clathrin adaptor complex AP‐2 by coincident detection of phosphatidylinositol 4,5‐bisphosphate [PI(4,5)P_2_] and sorting signals within cargo receptors that enables assembly of clathrin, that is, the outer coat layer, on the cytoplasmic face of the plasma membrane (McMahon & Boucrot, 2011; Kaksonen & Roux, 2018). Assembly of vesicle coats and coat‐associated proteins often comprising IDRs drives the acquisition of positive membrane curvature via hydrophobic insertion of amphipathic helices and/or the scaffolding of curved membranes by crescent‐shaped lipid binding domains (e.g., bin‐amphiphysin‐rvs [BAR] domains; Antonny, 2011; Stachowiak *et al*, 2013; Daumke *et al*, 2014; Kaksonen & Roux, 2018). As the vesicle bud matures, a negatively curved tubular membrane neck forms that eventually is separated from the donor membrane by protein‐catalyzed fission. In the case of endocytic vesicle formation, membrane fission is mediated by a GTP hydrolysis‐induced conformational change in oligomeric assemblies of the large GTPase dynamin. Vesicle release is tightly coupled to the disassembly of vesicle coats, thereby enabling the free uncoated vesicle to be transported (e.g., via motor proteins) to its target membrane (McMahon & Boucrot, 2011; Daumke *et al*, 2014; Kaksonen & Roux, 2018).

A topologically opposite type of vesicle formation away from the cytoplasm (e.g., into the lumen of endosomes or into the extracellular space) is catalyzed by the endosomal sorting complex required for transport (ESCRT) machinery. This pathway involves a spring‐loaded mechanism of membrane fission via spiral assembly of ESCRT‐III proteins on the cytoplasmic face of the budding membrane (Raiborg & Stenmark, 2009; Pfitzner *et al*, 2021). Yet, another distinct type of vesicle formation occurs in autophagy. This process involves the ordered assembly of core autophagy machinery proteins at sites where ATG9‐containing vesicles coalesce with ER membranes resulting in the formation of double membrane vesicles covered on either side with lipid‐conjugated LC3 family proteins. The content of autophagic vesicles is eventually delivered to lysosomes via membrane fusion for degradation (Vargas *et al*, 2023).

At the target membrane, incoming vesicles (e.g., secretory or endocytic vesicles, autophagosomes) are captured by Rab protein‐associated tethering factors (Zerial & McBride, 2001). Rabs together with PI lipids (e.g., PI(4,5)P_2_ at the plasma membrane) are codeterminants of organelle identity and membrane nanodomain organization and a conserved part of the machineries for vesicle docking and fusion (Fig 2B). Like vesicle coats, tethering factors are recruited to membranes via coincident binding to active small GTPases (i.e., GTP‐bound Rab proteins) and membrane lipids (e.g., PIs; Behnia & Munro, 2005; Di Paolo & De Camilli, 2006; Posor *et al*, 2022).

Tethering factors fall into two principle classes of unrelated proteins based on primary sequence and domain structure: Extended coiled‐coil proteins such as the Golgins or early endosome antigen 1 (EEA1) and multisubunit tethers, which can be further subdivided into the Complexes Associated with Tethering Containing Helical Rods (CATCHR) and Class C MTCs (multisubunit tethering complexes) subfamilies (Ungermann & Kummel, 2019). Examples of multisubunit tethering factors are the homomeric vacuole fusion and protein sorting (HOPS) and class C core vacuole endosome tethering (CORVET) complexes, the Exocyst complex (Ungermann & Kummel, 2019), and the assemblies formed by the presynaptic active zone protein Munc13 (discussed below; Sakamoto *et al*, 2018), which function in membrane fusion within the endolysosomal system and in regulated neuroexocytosis. Once vesicles are docked to their target, membrane fusion is driven by zippering of trans‐SNARE (soluble N‐ethylmaleimide‐sensitive factor attachment protein receptor) complexes present on either side of the fusing membranes (Fig 2B). The assembly of trans‐SNARE protein complexes that drive fusion requires chaperoning by S/M (Sec1/Munc18) proteins and is aided by tethers as exemplified by Munc13 at the presynaptic AZ (Sudhof & Rothman, 2009; Jahn & Fasshauer, 2012). Finally, postfusion cis‐SNARE complexes are disassembled via the activity of the N‐ethylmaleimide‐sensitive factor (NSF) ATPase and its adaptor protein α‐SNAP.

### Nonvesicular transport

Apart from exchanging signals, metabolites, and other molecules (e.g., proteins and lipids) by vesicular transport, organelles communicate and integrate their activities by nonvesicular transport at membrane contact sites (MCS). Membrane contact sites are sites of close membrane apposition (e.g., at distances that vary between about 20 to 60 nm), at which two or possibly more organelles physically interact but typically do not undergo fusion. In addition, intraorganelle contact sites between two regions of the same organelle (e.g., distinct parts of the ER or mitochondrial subcompartments) exist (Cohen *et al*, 2018; Wu *et al*, 2018; Prinz *et al*, 2020; Guillen‐Samander & De Camilli, 2023). A prominent hub for MCS formation is the ER, which forms a plethora of contacts with essentially all other eukaryotic cell organelles (Wu *et al*, 2018; Guillen‐Samander & De Camilli, 2023). Membrane contact sites considerably differ with respect to shape, composition, function, and dynamics. While some MCS are highly dynamic and form and dissociate on timescales of a few seconds, others such as the specialized contacts between the ER and the PM in muscle essentially persist for the entire lifetime of the cell or tissue. Among the known functions of MCS are calcium and lipid signaling and transport, metabolic channeling, membrane fission and fusion (e.g., of mitochondria; Wu *et al*, 2018; Guillen‐Samander & De Camilli, 2023), as well as organelle transport, dynamics, and reshaping (e.g., of the ER by MCS with endosomes; Jang *et al*, 2022).

Nonvesicular transport of signaling molecules, lipids, and metabolites at MCS (Fig 1) depends on membrane tethering proteins. Known tethers include transmembrane proteins (e.g., VAPs, VAMP‐associated proteins) in the ER and the PM (Saheki & De Camilli, 2017a; e.g., E‐Syts, extended synaptotagmins; Saheki & De Camilli, 2017b) as well as membrane‐associated factors recruited by coincident recognition of PIs and small GTPases (e.g., active Rabs) or other membrane‐bound proteins.

Well‐known examples of tethers at MCS are the complexes formed by ER‐localized VAP proteins with oxysterol‐binding protein 1 (OSBP1) at the trans‐Golgi network (TGN; Mesmin *et al*, 2013) and with oxysterol‐binding protein‐related proteins (ORPs) 5/8 at the PM (Chung *et al*, 2015). Moreover, the exchange of phosphatidylinositol for phosphatidic acid at PM‐ER MCS by the evolutionary conserved VAP‐binding protein Nir2 (RdgB in *Drosophila melanogaster*; Kim *et al*, 2015; Yadav *et al*, 2015) and the calcium‐regulated association of STIM1 in the ER with PM ORAI1 channels to mediate store‐operated calcium entry (Liou *et al*, 2005; Zhang *et al*, 2005; Feske *et al*, 2006) are of key importance for the maintenance and regulation of receptor signaling. Most MCS are held together by multiple, possibly partially redundant tethers. For example, at least seven different protein complexes are involved in tethering the cortical ER to the PM in yeast (Saheki & De Camilli, 2017a). Many of the tethering proteins at MCS serve additional roles, for example, as channels or transporters for lipids (e.g., OSBP, ORPs, E‐Syts, and ATG2; Saheki & De Camilli, 2017b; Leonzino *et al*, 2021) or calcium (e.g., STIM1‐ORAI1) or as enzymes (e.g., phosphatidylinositol 4‐phosphate [PI(4)P] hydrolysis by Sac1). Among the various types of factors found at MCS are proteins involved in membrane fission (e.g., the dynamin‐related protein DRP1; Kleele *et al*, 2021), membrane bending (e.g., the PX‐BAR domain protein SNX [sorting nexin] 2; Dong *et al*, 2016), organelle transport via motor proteins (e.g., the protrudin/FYCO1 complex at ER/late endosome MCS; Raiborg *et al*, 2015), vesicle budding (e.g., the ESCRT‐III protein IST1), or cell signaling (e.g., the protein phosphatase PTP1B).

## Common principles of vesicular and nonvesicular transport and the role of IDPs

Although vesicular and nonvesicular transport via MCS display apparent functional and morphological differences, work in recent years has uncovered a number of noteworthy molecular and conceptual similarities.

First, both vesicular and nonvesicular transport depend on identity determinants (e.g., small GTPases and PIs) that mark the respective membrane nanodomain to enable the specific recruitment and multimeric assembly of proteins (e.g., proteins from the cytoplasm or the cytoplasmic domains of membrane proteins) that serve as effectors in the pathway (Behnia & Munro, 2005; Di Paolo & De Camilli, 2006; Saheki & De Camilli, 2017a; Prinz *et al*, 2020; Posor *et al*, 2022). Depending on the process, these include coat proteins (Faini *et al*, 2013), membrane deforming proteins (e.g., BAR proteins and sorting nexins; (Daumke *et al*, 2014), or proteinaceous membrane tethers (Ungermann & Kummel, 2019). The latter may facilitate membrane apposition as in the case of MCS (Stefan *et al*, 2017; Prinz *et al*, 2020) or enable subsequent fusion by downstream factors as in SV exocytosis (Sudhof & Rothman, 2009; Haucke *et al*, 2011; Jahn & Fasshauer, 2012). As vesicle formation typically depends on membrane‐active (i.e., deforming) proteins (Daumke *et al*, 2014; Pfitzner *et al*, 2021), we predict that proteins, particularly IDPs, involved in nonvesicular transport at MCS in many cases may also be membrane‐active. Experimental evidence for this proposal is lacking at this time. Second, the pivotal role of PIs and small GTPases necessitates mechanisms for the spatiotemporally controlled writing and erasing of membrane identity on the nanoscale (Behnia & Munro, 2005; Posor *et al*, 2022). Consistently, overlapping sets of PI kinases and phosphatases (Di Paolo & De Camilli, 2006; Schink *et al*, 2016; Posor *et al*, 2022) as well as guanine nucleotide exchange factors (GEFs) and GTPase‐activating proteins (GAPs; Lamber *et al*, 2019) have been implicated in vesicle formation, fission, and fusion (Fig 2) and in MCS assembly (Fig 1) to facilitate nonvesicular transport. Finally, the dynamic nature of vesicular and nonvesicular transport necessitates that the underlying biochemical network comprises low‐affinity protein–protein and protein–lipid interactions that are considerably strengthened when restricted to the two‐dimensional space of membrane interfaces (Kalappurakkal *et al*, 2020).

**Figure 3:**
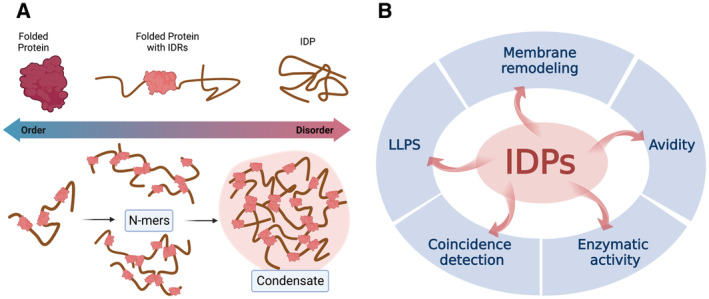
Principle modes of operation and functional roles of IDPs (A) The continuum of intrinsic disorder. Top, proteins can be described either as folded, as having a modular architecture with both folded and intrinsically disordered regions (IDRs), or as true IDPs, in which the entire polypeptide contains little or no stable structure. Bottom, multivalent proteins can polymerize via homotypic and/or heterotypic association (i.e., N‐mers) into condensates. (B) Principle functionalities promoted by IDPs. Created with BioRender.com.

To cope with the geometrical constraints (e.g., the varying distance of two organelles undergoing MCS formation or docking and fusion) and to enable metastability of the system (McMahon & Boucrot, 2011; Faini *et al*, 2013), many of the proteins involved in vesicular and nonvesicular transport display a pearls‐on‐a‐string‐like architecture, in which stably folded domains (e.g., an enzymatic core) are paired with long (> 30 amino acids in length, also referred to as long disordered regions) IDR segments harboring low‐affinity (Kd of 1–100 μM) protein or lipid interactions motifs (Fig 3A). Intrinsically disordered proteins may constitute more than 30% of the eukaryotic proteome (Colak *et al*, 2013; Deiana *et al*, 2019) and can be predicted by computational and machine‐learning‐based methods (> 40 algorithms exist; e.g., Jones & Cozzetto, 2015; Liu *et al*, 2019) based on amino acid sequence complexity and flexibility, bias toward certain amino acids and secondary structure information. These methods are often used in conjunction with experimental data derived from nuclear magnetic resonance (NMR) spectroscopy, protein X‐ray crystallography, electron paramagnetic resonance (EPR) spectroscopy, circular dichroism spectroscopy, small‐angle X‐ray scattering, or single‐molecule fluorescence resonance energy transfer (see Chowdhury *et al*, 2023 and references within) to generate IDR/IDP databases. Typically, IDRs display a relatively low proportion of hydrophobic and bulky aromatic residues, which usually form the hydrophobic core of globular proteins (Jamecna & Antonny, 2021). Instead, they are enriched in polar or charged residues and in secondary structure‐disrupting proline residues (Martin & Holehouse, 2020). Intrinsically disordered regions do not fold into a fixed three‐dimensional structure but instead exist in a heterogeneous ensemble of conformations (Fig 3A). Intrinsically disordered regions have recently entered mainstream cell biology in the context of liquid–liquid phase separation (LLPS; Martin & Holehouse, 2020). Clearly, IDRs are frequently found to be enriched in phase‐separated compartments, and indeed recent evidence suggests that IDRs in addition to displaying conserved site‐specific interaction motifs contribute to the formation of mesoscale protein assemblies via LLPS into protein condensates. These condensates often exhibit a low degree of internal order and could operate over a broad range of stoichiometries between the protein molecules that form the assembly. An important functional distinction can be made between driver proteins, which spontaneously phase separate, and client proteins that require additional components—typically driver proteins—to phase separate (Banani *et al*, 2017).

While the presence of an IDR in a protein is frequently assumed to be diagnostic of its ability to phase separate, it is important to note that although some disordered regions will robustly drive phase separation, others will not (Martin & Holehouse, 2020). Likely, instead of disorder *per se*, multivalency (also referred to as avidity) may be at the core of IDP function in biology (Fig 3A). Often, multivalent IDR‐containing proteins adopt states characterized by autocontacts that are biologically inactive. Upon the arrival of specific signals or physiological cues these proteins might “open” up and exploit their multivalent character to promote homo‐ and/or heterotypic protein assembly (Fig 3A). In this context, multivalent IDPs may also serve as co‐incidence detectors (Fig 3B) that integrate distinct signals into coherent biological action (McMahon & Boucrot, 2011; Musacchio, 2022). Among the numerous examples of IDPs in vesicular and nonvesicular transport (i.e., membrane remodeling) are components of vesicle coats (e.g., AP clathrin adaptors, SNXs, Sec31/COPII), tethers (e.g., Munc13, Golgins and related Rab effectors such as EEA1, VAP‐A/B), or enzymes (i.e., GEFs, GAPs for small GTPases, PI phosphatases, and their complexes).

As indicated above, condensate formation has been found to be regulated by various cellular signals including changes in local concentrations, posttranslational modifications, energy‐consuming processes, and biomolecular interactions, for example, with small molecules including lipids (Garcia‐Cabau & Salvatella, 2021). By now, LLPS of membrane‐associated IDPs has been implicated in the clustering of synaptic vesicles for temporally controlled fusion and storage (e.g., Milovanovic *et al*, 2018; Pechstein *et al*, 2020; Sansevrino *et al*, 2023), the formation of autophagosomes (Fujioka *et al*, 2020; Agudo‐Canalejo *et al*, 2021) and endocytic vesicles (Day *et al*, 2021), and in nonvesicular lipid transport at MCS between the ER and the TGN mediated by VAP proteins (Jamecna & Antonny, 2021; Subra *et al*, 2023).

In the following, we will discuss specific examples that illustrate how this property of IDPs may facilitate both vesicular and nonvesicular transport.

## Membrane‐associated IDPs in vesicular transport

A well‐studied example of vesicular transport is the formation of clathrin‐coated endocytic vesicles at the PM. Clathrin‐mediated endocytosis (CME) produces spherical vesicles of 80–150 nm in diameter that are covered by regular hexagonal and pentagonal arrays of stably folded clathrin triskelia, whose formation is governed by a complex assembly pathway involving specific coincident recognition of endocytic proteins and membrane lipids (Di Paolo & De Camilli, 2006; McMahon & Boucrot, 2011; Kaksonen & Roux, 2018; Posor *et al*, 2022). The vast majority of endocytic proteins (e.g., Epsins, CALM/AP180, Eps15, FCHo1/2, Intersectin1/2, AP2, Stonin2, Amphiphysin1/2) contain IDRs, resulting in a “fuzzy” endocytic protein interaction network (McMahon & Boucrot, 2011; Kaksonen & Roux, 2018; Schiano Lomoriello *et al*, 2022). The IDRs in endocytic proteins may aid endocytic vesicle assembly through distinct mechanisms. Short IDRs found, for example, in the N terminus of Epsin, upon binding to charged lipids can undergo folding into amphipathic helices that promote PM curvature acquisition (Ford *et al*, 2002). Additionally, the long IDRs that form the C‐terminal domains of Epsin or AP180 appear to exhibit sterically repulsive entropic forces that further promote PM bending to aid endocytic vesicle formation (Busch *et al*, 2015; Yuan *et al*, 2023). Moreover, it has recently been shown that the early‐acting endocytic initiator proteins Eps15 (Ede1 in yeast) and FCHo use weak, multivalent interactions involving IDRs to form liquid‐like assemblies that promote the recruitment of downstream interactors and, thereby, facilitate endocytic vesicle formation (Day *et al*, 2021). This mechanism appears to be evolutionary conserved from yeast to man (Kozak & Kaksonen, 2022). Intersectin, an Eps15‐binding early‐acting endocytic protein with a prominent role at synapses and in cell signaling has been shown to coalesce into LLPS in the presence of phase‐separating driver proteins such as Synapsin (Milovanovic *et al*, 2018; Pechstein *et al*, 2020). It is tempting to speculate that subsequent steps of endocytic vesicle formation may also involve LLPS of distinct sets of proteins (e.g., CALM, HIP1R, or Epsins), possibly resulting in multiphase condensates that couple endocytic protein nanoscale localization to distinct steps in CME (Witkowska & Haucke, 2021).

Protein condensate formation via LLPS has also been demonstrated to play a role in the formation of autophagic vesicles. In yeast cells starved for nitrogen, autophagy is initiated at distinct intracellular sites via co‐condensation of the kinase autophagy‐related protein 1 (Atg1), the homolog of ULK1/2 in mammals, with its binding partners Atg13, another IDR protein, and the Atg17‐Atg29‐Atg31 complex into a large multimeric protein assembly (Yamamoto *et al*, 2016). This assembly interacts with endomembranes to facilitate the subsequent steps of autophagic double membrane vesicle formation. In mammalian cells, it has been suggested that LLPS of p62, a protein that serves as a receptor for degradation‐prone protein aggregates (i.e., aggrephagy), creates a template for a wetting process that enables the assembly of autophagic vesicles on the surface of the p62 condensate (Agudo‐Canalejo *et al*, 2021). Intrinsically disordered proteins have also been implicated in ER‐phagy, the specific autophagy of ER membranes. Here, the long extended IDR of the ER‐phagy receptor TEX264 has been shown to bridge between the ER (i.e., the autophagic cargo) and the autophagosomal vesicle membrane (Chino *et al*, 2019).

These examples illustrate how IDRs can support and control vesicular transport in multiple ways by regulating the assembly and physicochemical properties of multivalent protein complexes involved in vesicle formation. We predict that the IDRs found in many other vesicular transport proteins play similarly important roles in the control of vesicle formation, docking, and fusion.

## Membrane‐associated IDPs in nonvesicular lipid transport and MCS assembly

**Figure 4:**
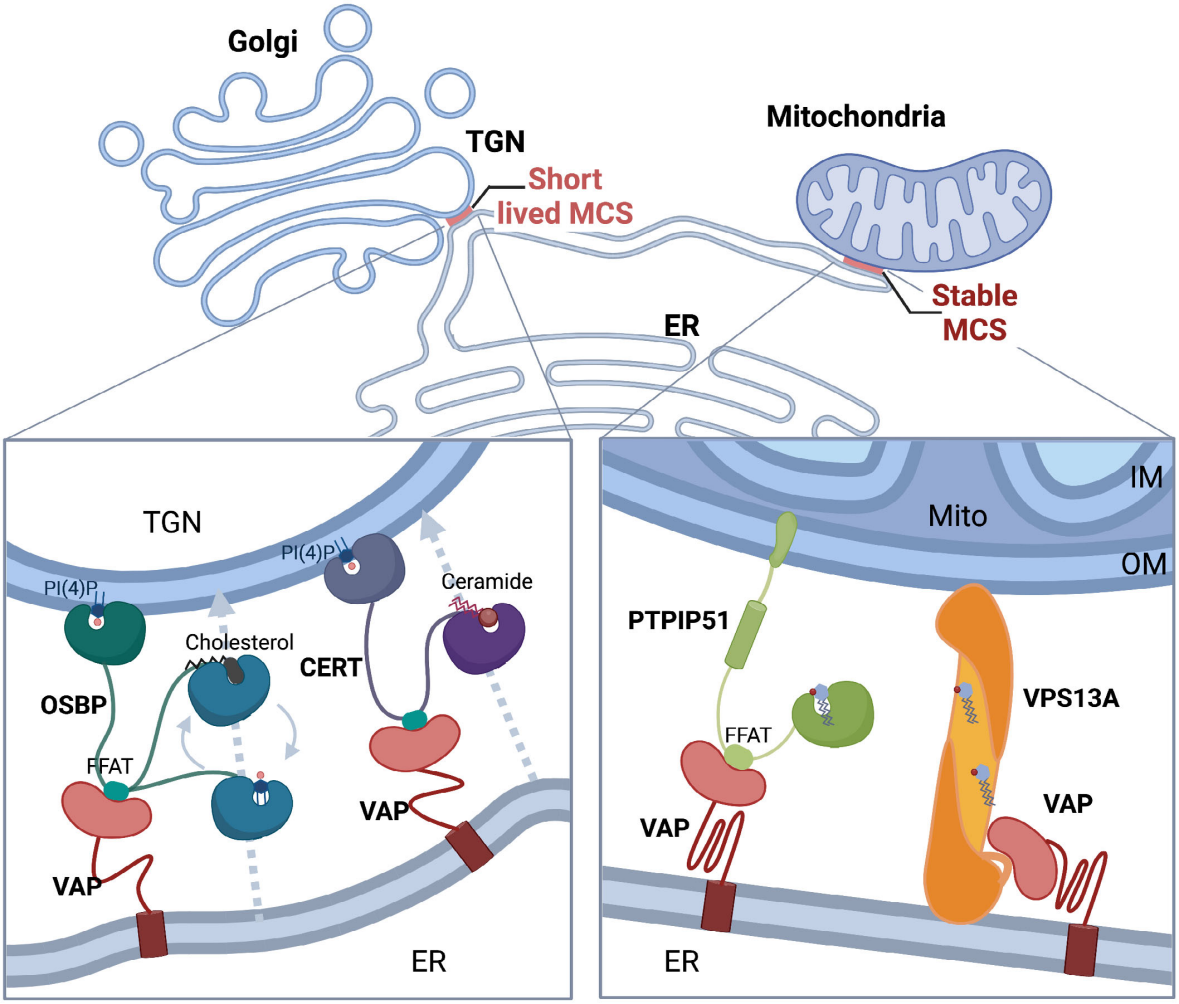
Model showing how the IDR enables VAP‐A to engage in distinct contacts between the ER and the TGN (left) or mitochondria (right) Short‐lived MCS with the TGN require VAP‐A to explore a large conformational space via its flexible linkers. Stable MCS between the ER and mitochondria allow VAP‐A to be in a more rigid, compact conformation. See review text for more information. Created with BioRender.com.

Nonvesicular lipid transport at MCS is mediated by lipid transfer proteins (LTPs), which frequently contain both structured domains and IDRs (Jamecna & Antonny, 2021). Recent data suggest that IDRs affect nonvesicular lipid transport by (i) acting as flexible tethers between membranes, (ii) by serving as entropic barriers that regulate protein density and the spatial arrangement of the tether, and (iii) by controlling the catalytic lipid‐transfer domains of LTPs (Jamecna *et al*, 2019; Jamecna & Antonny, 2021). Well‐studied examples of LTPs are the various members of the oxysterol‐binding protein‐related proteins (ORPs; Chung *et al*, 2015; Stefan *et al*, 2017; Wu *et al*, 2018; Peretti *et al*, 2019; Subra *et al*, 2023). Oxysterol‐binding protein‐related proteins typically harbor an N‐terminal IDR of about 50–140 amino acids upstream of their PH domain. In case of ORP5 and ORP8, that is, ORPs that localize to ER‐PM MCS, it was demonstrated that the N‐terminal IDRs contribute to PM targeting by electrostatic interactions, thereby synergizing with the PI‐binding PH domain (Chung *et al*, 2015). Biochemical and cellular experiments have suggested that the N‐terminal IDR of OSBP, the founding member of the ORP family, can act as an entropic shield that serves a dual function: It prevents the homotypic symmetrical apposition of two Golgi membranes via the PI(4)P‐binding PH domain of OSBP dimers and counteracts protein crowding to facilitate the dynamic tethering of TGN membranes to the ER (Jamecna *et al*, 2019). Recent data have uncovered yet another function of IDRs in nonvesicular lipid transport at MCS by controlling the partitioning of multifunctional tethers between distinct MCS. Specifically, it was demonstrated that the IDR of ER‐localized VAP proteins is required for its association with OSBP and CERT to enable lipid exchange at ER‐Golgi MCS, but dispensable for VAP‐mediated MCS with PTPIP51 and Vps13A at mitochondria (Subra *et al*, 2023; Fig 4). Hence, in case of VAP, the IDR serves to adjust the geometry to the organization and dynamics of the respective MCS. One might hypothesize that modulation of such IDR‐mediated mechanisms (e.g., via posttranslational modifications) enables cells to channel nonvesicular lipid transport according to physiological needs.

Many other LTPs such as the mammalian lipid transporter TMEM24 (also called C2CD2L; Lees *et al*, 2017) and the yeast ER membrane protein Ist2, proteins enriched at MCS between the ER and the PM, have been shown to contain IDRs of different length (Wong *et al*, 2021) and may thereby contribute to the regulation of the distance between the two apposed membranes. Another function of IDRs within proteins involved in nonvesicular lipid transport is exemplified by the autophagy protein ATG2. Here, an amphipathic helix in the C‐terminal region of ATG2 binds to membranes and facilitates ATG18 binding to PI(3)P to target the ATG2‐ATG18 complex to the preautophagosomal structure to enable autophagic vesicle formation (Kotani *et al*, 2018). Whether IDR‐containing proteins at MCS, possibly depending on cell state and conditions, can undergo phase separation and whether and how this may contribute to the regulation of MCS dynamics and the nonvesicular transport of lipids and other molecules is an exciting open question (Jamecna & Antonny, 2021).

## Membrane‐associated IDPs in presynaptic neurotransmission

**Figure 5:**
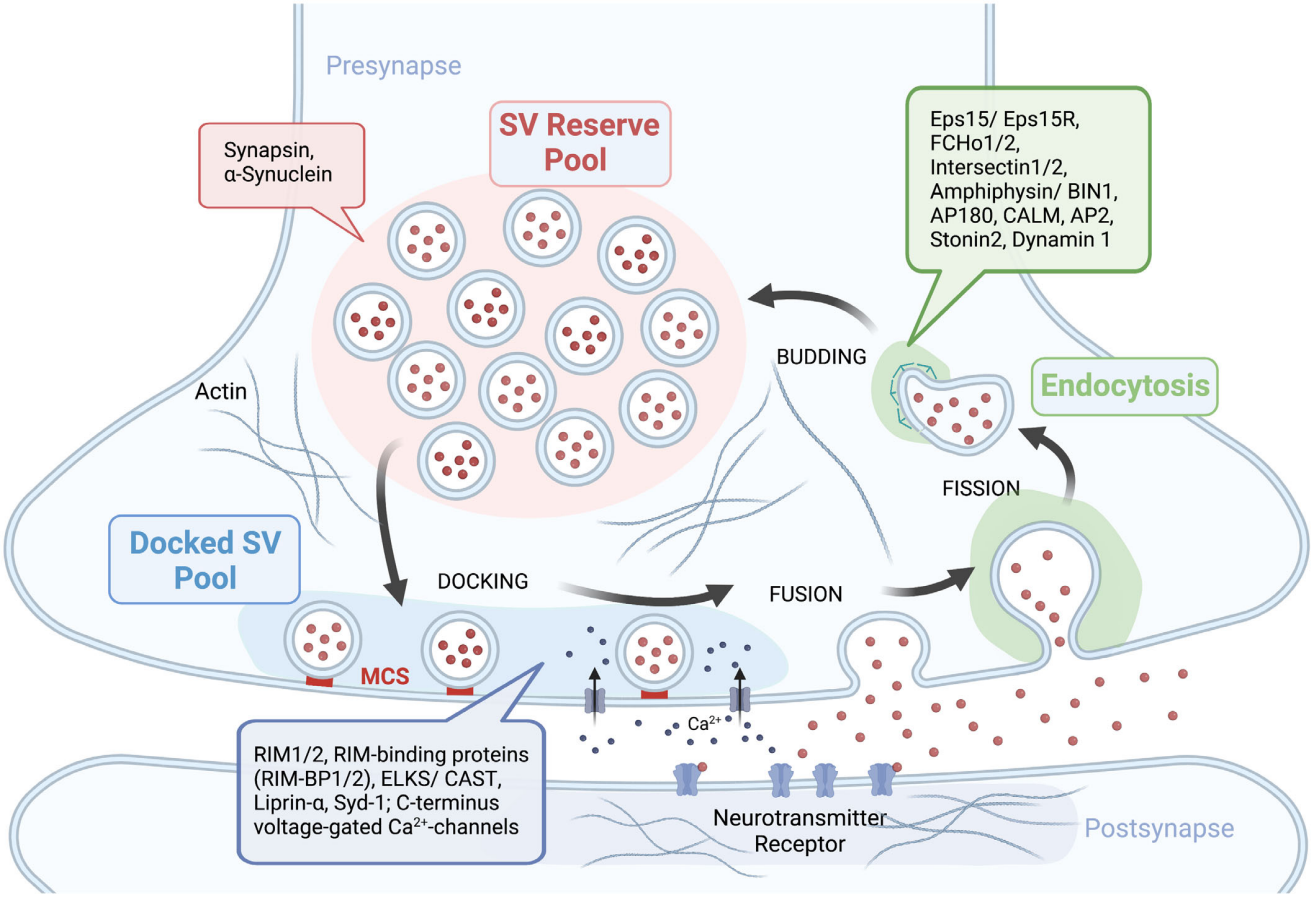
IDPs orchestrating presynaptic neurotransmission and vesicle cycling SVs are organized into a docked SV pool close to the active zone (AZ) and a reserve pool that is controlled by Synapsin and α‐Synuclein. AZ proteins such as RIM1/2, RIM‐binding proteins, ELKS/CAST, Liprin‐α, Syd‐1, and the C terminus of voltage‐gated Ca^2+^ channels may form a protein condensate that facilitates fast neurotransmission. Endocytosis of SV membranes and SV reformation via membrane fission and budding processes is mediated by IDR‐containing endocytic proteins such as Eps15/Eps15R, FCHo1/2, Intersectin 1/2, Amphiphysin/BIN1, AP180, CALM, AP2, Stonin2, and Dynamin 1 that may form endocytic protein condensates. Created with BioRender.com.

Presynaptic neurotransmitter release is dominated by the synaptic vesicle (SV) cycle and entails the biogenesis, fusion, recycling, reformation, or turnover of SVs (Haucke *et al*, 2011)—processes that involve bulk movement of membrane lipids and proteins (Binotti *et al*, 2016; Fig 5). Synaptic vesicle undergo regulated membrane fusion and endocytosis at high speed (e.g., at sub‐millisecond timescale for excocytosis), way faster than at most other cellular locales (Sudhof & Rothman, 2009; Haucke *et al*, 2011; Jahn & Fasshauer, 2012). At the same time, robust performance is key to maintain the functionality of the synapse for circuit and ultimately brain function. Moreover, the system is highly plastic across timescales ranging from milliseconds (short‐term) to hours (long‐term) and more, as neurotransmission is adapted to internal or external cues (e.g., learning). Indeed, fast, use‐dependent remodeling of the presynaptic protein architecture (“presynaptic plasticity”) is considered to be of major importance for nervous system function, for example, for acute information filtering and sensation, long‐term memory, or sleep homeostasis (Monday *et al*, 2018; Huang & Sigrist, 2021; Shahoha *et al*, 2022). In order to be able to quickly boost SV release, a large proportion of SVs typically is sequestered within the so‐called reserve pool. Reserve pool SVs can be transferred and finally docked at the neuronal AZ plasma membrane by forming a metastable MCS (i.e., docked SVs) that enables millisecond fusion in response to an incoming action potential. Physical docking is intimately intertwined with biochemical “priming” of SVs that serves as an essential precondition for subsequent membrane fusion via tightly assembled SNARE complexes. Ultimately, SV fusion is triggered by the action potential evoked nanodomain Ca^2+^ entry via voltage‐gated Ca^2+^ channels (Sudhof & Rothman, 2009; Jahn & Fasshauer, 2012). Voltage‐gated Ca^2+^ channels are positioned with high nanoscale precision relative to docked SVs that harbor the exocytic Ca^2+^‐sensing transmembrane protein Synaptotagmin. Following fusion, exocytosed SV proteins and lipids are internalized by kinetically distinct forms of endocytosis, typically involving dynamin‐mediated membrane fission (Kononenko & Haucke, 2015; Chanaday *et al*, 2019). The key functions of this compensatory endocytosis are to restore PM area and tension and to recycle SV proteins and lipids for SV reformation via clathrin‐mediated vesicle budding to refill the SV pool. Many, if not all, of these steps (Fig 5) are regulated by actin dynamics (Wu & Chan, 2022) and by IDPs, possibly involving LLPS (Wu *et al*, 2020; Hayashi *et al*, 2021; Lautenschlager, 2022). Bioinformatic analysis of the presynaptic protein repertoire, for example proteins that control the SV cycle, has revealed a particularly high abundance of IDPs (Lautenschlager, 2022). These include many scaffold proteins of the AZ scaffold (discussed in detail below), factors involved in SV docking or priming (i.e., Munc13‐family) as well as endocytic proteins (e.g., FCHo1/2, Intersectin1/2, Amphiphysin/BIN1, AP180, CALM, AP2, and Stonin2) including Eps15/Eps15R (Day *et al*, 2021) and Dynamin 1 (Imoto *et al*, 2022). The overrepresentation of IDPs at the presynapse may relate to two of the mentioned key features of neurotransmission (Fig 5): The extremely high speed of SV exo‐ and endocytosis and the remarkable spatiotemporal precision with which SV docking, fusion and endocytic recycling occur. Plastic assembly and dynamics of physically extended IDP‐based protein networks may be required to orchestrate the required crosstalk between the distinct functional subsystems of the presynapse, for example, the machineries for SV docking at the AZ (Acuna *et al*, 2016; Wu *et al*, 2019, 2021), clustering of SVs within the reserve pool (Milovanovic *et al*, 2018; Pechstein *et al*, 2020), and endocytic membrane retrieval at sites that surround the AZ membrane (Day *et al*, 2021; Witkowska & Haucke, 2021).

### The active zone and the docked pool of SVs

The so‐called AZ for SV fusion harbors an evolutionary conserved set of physically extended IDPs that comprise the multidomain RIM proteins (RIM1/2), RIM‐binding proteins (RIM‐BP1/2), ELKS/CAST (orthologs of BRP in *D. melanogaster*), Liprin‐α, and Syd‐1. A major function of AZ proteins is to direct SVs to defined “release sites,” which in turn are spatially and, likely, biochemically coupled to presynaptic voltage‐gated Ca^2+^ channels (Sakamoto *et al*, 2018; Fig 5). Recent biochemical data suggest that presynaptic scaffold proteins via their extended IDRs might form ordered assemblies and display the propensity to undergo LLPS. RIM and RIM‐BP, when mixed *in vitro*, can autonomously form condensed assemblies via LLPS. Such RIM/RIM‐BP condensates have been demonstrated to enable the clustering of the cytosolic tails of voltage‐gated Ca^2+^‐channels tethered to lipid membranes (Wu *et al*, 2021). RIM/RIM‐BP condensates might thus serve as “molecular rulers” that determine the positioning of SV docking and biochemical priming with respect to voltage‐gated Ca^2+^ channels within the AZ membrane nanodomains organizing neurotransmitter release. In support of this, phase‐separated RIM/RIM‐BP assemblies have recently been shown (Wu *et al*, 2021) to be able to capture negatively charged liposomes or native SVs purified from brain on the surface of giant unilamellar vesicle (GUV) membranes harboring the clustered cytoplasmic IDRs of voltage‐gated Ca^2+^‐channels. This LLPS‐based mechanism for organizing the highly specialized MCS between release‐ready SVs and Ca^2+^‐channels within the AZ membrane appears to be evolutionary conserved. In *D. melanogaster* RIM‐BP cooperates with the large AZ scaffold protein BRP, the ortholog of ELKS/CAST proteins in mammals and *Caenorhabditis elegans*, to cluster voltage‐gated Ca^2+^‐channels (Petzoldt *et al*, 2020) and couple them to release‐ready SVs associated with BRP at the so‐called electron‐dense body AZ scaffold (Hallermann *et al*, 2010). Loss of BRP results in fast use‐dependent synaptic depression caused by defective recruitment of primed SVs to Ca^2+^‐channels at the AZ release site, a phenotype similar to that observed at rodent hair cell synapses devoid of RIM‐BP2 (Krinner *et al*, 2017). Conversely, sustained presynaptic potentiation is driven by increased recruitment of voltage‐gated Ca^2+^ channels to the AZ and a concomitant compaction of BRP nanoclusters and the associated channels, possibly reflecting phase transitions of the AZ scaffold (Ghelani *et al*, 2023).

Studies in *C. elegans* have shown that the ability of AZ scaffolds to undergo LLPS via multivalent interactions between Liprin‐α and the N‐terminal domain of ELKS/CAST (i.e., the ortholog of BRP) underlies also presynapse assembly during development. Liprin‐α and ELKS/CAST initially form highly dynamic liquid condensates (Fig 5) during early developmental stages, which then mature into more rigid condensates characteristic of the mature AZ (McDonald *et al*, 2021).

### The SV reserve pool

The docked SV pool is physically, functionally, and molecularly distinct from the SV reserve pool (Fig 5). Phase‐separating AZ IDPs (for example, ELKS/CAST/BRP, Munc13, RIM‐BPs) are likely largely absent from the SV reserve pool and disruption of AZ scaffolds selectively impairs the docked pool of SVs (Acuna *et al*, 2016; Wang *et al*, 2016). Instead, as mentioned above, reserve pool SVs are marked by Synapsin, an abundant IDP that reversibly binds to highly curved membranes such as SVs (Krabben *et al*, 2011) and can undergo LLPS and thereby form vesicle assemblies akin to those observed at synapses *in situ* (Milovanovic *et al*, 2018). Consistently, perturbation or loss of Synapsin selectively impacts the SV reserve pool, whereas the docked SV pool at the AZ remains unchanged (Pieribone *et al*, 1995; Rosahl *et al*, 1995). Interestingly, the Parkinson's disease‐associated IDP α‐Synuclein can mix with Synapsin phases, and the presence of SVs enhances the rate of Synapsin condensation, suggesting that SVs act as catalysts for the formation of Synapsin/α‐Synuclein co‐condensates (Hoffmann *et al*, 2021). Liquid–liquid phase separation thus likely underlies the ability of Synapsin to cluster SVs within the reserve pool at native synapses in brain.

These data favor a model according to which the distinct pools of SVs comprise distinct condensates of the same organelles (i.e., SVs) and thereby contribute to the spatial organization of the presynapse on the nanoscale.

### Regulation of the SV cycle and synapse function

The emerging role of IDPs and their ability to undergo LLPS in presynapse formation and function poses the important question by which principles IDPs drive assembly and how their association with membranes is regulated. Synapsin, the IDP that marks and drives SV reserve pool organization (Rosahl *et al*, 1995; Milovanovic *et al*, 2018; Pechstein *et al*, 2020), has long been known to undergo activity‐dependent phosphoregulation. Phosphorylation of Synapsin by presynaptic kinases such as Cdk5 facilitates its dissociation from SV membranes, thereby enabling the migration of SVs to the docked vesicle pool at the AZ (Verstegen *et al*, 2014). Similarly, ELKS/CAST/BRP and Liprin‐α3 have been shown to be the substrates of kinases (i.e., SRPK79D for BRP and PKC for Liprin‐α3) that can reversibly regulate their assembly and, thereby, control AZ scaffold protein transport, assembly, and ultimately neurotransmission (Driller *et al*, 2019; Emperador‐Melero *et al*, 2021).

Aside from kinases and phosphatases Rab GTPases have been shown to play major roles in presynapse assembly and function. Rab3, Rab27, and Rab35 have been suggested to regulate distinct steps of SV cycling and/or the endosomal sorting of SV proteins via vesicular transport (Schluter *et al*, 2006; Binotti *et al*, 2016). Many of their GEFs, GAPs and effectors such as RIM and Rabphilin, are IDPs (Wu *et al*, 2020; Hayashi *et al*, 2021; Lautenschlager, 2022), opening the possibility that Rabs indeed might acts as molecular switches that reversibly direct LLPS to plastically change synapse function.

Recent work (Imoto *et al*, 2022) has shown that a phase‐separating splice variant of the membrane‐fissioning GTPase Dynamin 1 (called Dynamin 1xA) is pre‐recruited to endocytic sites via the FBAR‐SH3 domain protein Syndapin. This mechanism has been postulated to enable ultrafast endocytosis in response to single action potentials in hippocampal neurons. It is conceivable, if not likely, that endocytic protein condensate formation (Day *et al*, 2021; Witkowska & Haucke, 2021) also is of importance during SV reformation via clathrin/AP2‐mediated budding from endosome‐like vacuoles within presynaptic nerve terminals (Fig 5).

Similar principles may apply to membrane dynamics at the postsynaptic compartment, where IDPs that comprise the postsynaptic density (Zeng *et al*, 2016) and AMPA‐type glutamate receptors (Zeng *et al*, 2019) have been shown or suggested to undergo LLPS.

## IDP dysfunction in genetic disorders of vesicular and nonvesicular membrane dynamics

Over the last decades, mutations in more than 300 genes implicated in vesicular and nonvesicular membrane dynamics have been identified to underlie human diseases, many of them encoding IDPs. These monogenic diseases essentially can affect any type of tissue or organ (e.g., neurological disease, metabolic disorders, liver, muscular, and renal disorders, diabetes, cancer) and most frequently arise from loss‐of‐function mutations, although toxic gain‐of‐function mutants have been reported as well. Monogenic diseases of IDPs involved in membrane dynamics may be developmental in nature or only manifest during adulthood or in aging, often with prominent phenotypes in the brain. We focus here on alluding to general principles and some specific examples of genetic disorders of membrane dynamics caused by mutations in IDPs (Table 1). For a systematic overview, the reader is referred to excellent recent reviews (Sanger *et al*, 2019; Yarwood *et al*, 2020; Garcia‐Cazorla *et al*, 2022). In many cases, the identified mutations alter the expression level, stability, or the assembly properties of the encoded IDP (see the example of Tau) or, in case of enzymes such as MTM1 or MTMR2, their enzymatic activity, rather than affecting the IDR itself. Hence, the fact that many of the disease genes implicated in vesicular and nonvesicular membrane dynamics encode IDPs may reflect the importance of IDPs within disease‐relevant pathways rather than a specific function of the IDRs themselves. In fact, mutations within IDRs often may be tolerated comparably well as they lie within poorly structured regions of the protein.

**Table 1:** Examples of genetic disorders involving IDR‐containing proteins implicated in vesicular transport.

Gene	Protein function	Transport process	Known synaptic function	Disease	Ref.
SPG11 (Spatacsin)	IDP associated with AP5 complex	Autophagy/lysosome function	No	Ataxia: Autosomal recessive hereditary spastic paraplegia/paraparesis (HSP) & Amyotrophic lateral sclerosis (ALS)	Orlacchio *et al* (2010), Stevanin *et al* (2007), Vantaggiato *et al* (2019)
ZFYVE26 (Spastizin)	IDP associated with AP5 complex	Autophagy/lysosome function	No	Ataxia: Autosomal recessive hereditary spastic paraplegia/paraparesis (HSP)	Vantaggiato *et al* (2019)
AP4B1 AP4E1 AP4M1 AP4S1	Subunits of AP4 complex	TGN/endosomal sorting of autophagy protein ATG9	Yes	Ataxia: Hereditary spastic paraplegia/paraparesis (HSP)	Sanger *et al* (2019)
TANGO2	Scaffold protein at the ER with possible additional role at mitochondria	ER‐to‐Golgi‐transport; possibly also mitochondrial function	No	Ataxia: Hereditary spastic paraplegia/paraparesis (HSP) (SPG6) with encephalopathy and seizures; rhabdomyolysis, lactic acidosis, hypoglycemia; cardiac arrhythmias	Berat *et al* (2021)
AP1G1 AP1S1 AP1S2	Subunits of AP1 complex	TGN/endosomal sorting	Yes	Microcephaly (all); Intellectual disability & Epilepsy (AP1G1) MEDNIK syndrome (AP1S1) Pettigrew Syndrome (AP1S2)	Usmani *et al* (2021)
TBC1D20	Rab1‐GAP	Golgi traffic & integrity	No	Microcephaly: Warburg Microsyndrome cataracts; male Infertility	Liegel *et al* (2013)
AP3B1 AP3D1	Subunits of AP3 complex	Endosomal sorting	Yes	Early infantile epileptic encephalopathy; Hermansky‐Pudlak Syndrome 2 (albinism, immunodeficiency)	Dell'Angelica (2009), Huizing *et al* (2020)
AP2M1	Subunit of AP2 complex	Endocytosis	Yes	Developmental and epileptic encephalopathy	Helbig *et al* (2019)
ARH	Endocytic clathrin adaptor for LDL receptor	Endocytosis	No	Autosomal recessive hypercholesterolemia	Garcia *et al* (2001)
SYNJ1	Phosphatidylinositol 4′‐ and 5′‐phosphatase	Endocytosis; synaptic vesicle cycling; autophagy	Yes	Parkinson's disease; Alzheimer's disease	Krebs *et al* (2013), Quadri *et al* (2013)
SAC2/INPP5F	Phosphatidylinositol 4′‐phosphatase	Endocytic vesicle dynamics	Yes	Parkinson's disease	Cao *et al* (2020)
BIN1	Endocytic adaptor amphiphysin 2	Endocytosis; synaptic vesicle cycling	Yes	Alzheimer's disease	Lee *et al* (2002), Nicot *et al* (2007)
PICALM	Endocytic clathrin adaptor CALM	Endocytosis of postsynaptic AMPA receptors	Yes	Alzheimer's disease	Harold *et al* (2009), Jun *et al* (2010)
MTM1	Phosphatidylinositol 3′‐phosphatase	Membrane dynamics at endosomes; autophagy	No	Centronuclear myopathy	Amoasii *et al* (2012), Laporte *et al* (2000)
MTMR14	Phosphatidylinositol 3′‐phosphatase MTMR14	Membrane dynamics at endosomes; autophagy	No	Centronuclear myopathy	Amoasii *et al* (2012)
MTMR2	3′‐phosphatase MTMR2	Membrane dynamics at endolysosomes	No	Charcot–Marie‐Tooth Type 4B1	Amoasii *et al* (2012)
MTMR5 (SBF1)	Noncatalytic subunit of 3′‐phosphatase MTMR2	Membrane dynamics at endolysosomes	No	Charcot–Marie‐Tooth Type 4B3	Azzedine *et al* (2003)
MTMR13 (SBF2)	Noncatalytic subunit of 3′‐phosphatase MTMR2	Membrane dynamics at endolysosomes	No	Charcot–Marie‐Tooth Type 4B2	Azzedine *et al* (2003)
OCRL	Phosphatidylinositol 5′‐phosphatase	Membrane dynamics at endosomes	No	Oculocerebrorenal syndrome of Lowe; Dent's disease	Hoopes Jr *et al* (2005)

### Genetic disorders of IDPs involved in vesicular transport

Mutations in the endosomal clathrin adaptor complex AP5, a protein comprising a central α‐solenoid with two long flexible IDRs harboring protein–protein interacting appendage domains, and the associated large IDPs SPG11/Spatacsin (Stevanin *et al*, 2007) and ZFYVE26/Spastizin (Vantaggiato *et al*, 2019) cause autosomal‐recessive spastic paraplegia/paraparesis (HSP), a movement disorder that manifests in progressive loss of lower limb movement control (Table 1). Mutations in SPG11/Spatacsin are also implicated in amyotrophic lateral sclerosis (ALS; type 5; Orlacchio *et al*, 2010), another type of movement disorder caused by motoneuron degeneration. AP5, SPG11/Spatacsin and ZFYVE26/Spastizin have been linked to autophagy and vesicular membrane dynamics within the endolysosomal system (Toupenet Marchesi *et al*, 2021). A different form of hereditary spastic paraplegia is associated with loss‐of‐function mutations in the closely related clathrin adaptor complex AP4 (Sanger *et al*, 2019) that indirectly affects autophagy by regulating vesicular traffic of the transmembrane protein ATG9. Mutations in the IDR‐containing β and δ subunits of the endosomal AP3 complex have been linked to hypopigmentation in Hermansky–Pudlak syndrome (Dell'Angelica, 2009; Huizing *et al*, 2020). Mutations in the μ subunit of the endocytic AP2 complex have been found to underlie developmental epileptic encephaly in children (Helbig *et al*, 2019). Some neurodevelopmental disorders with microcephaly have been found to be caused by mutations in genes encoding the clathrin adaptor complex AP1 (Usmani *et al*, 2021), a critical mediator of clathrin‐coated vesicle formation at the trans‐Golgi network (TGN)/endosome interface, the COPII vesicle coat protein Sec23, or the COPII‐associated IDP TANGO2 (Bethune & Wieland, 2018). TANGO2 variants result in a complex disease phenotype consisting of encephalopathy, seizures, rhabdomyolysis, lactic acidosis, hypoglycemia, and cardiac arrhythmias (Berat *et al*, 2021). A further prominent example is the ARH gene, which encodes an endocytic PI lipid‐binding IDP dedicated to the endocytic sorting of low‐density lipoprotein receptor. Mutation of ARH causes an autosomal recessive form of hypercholesterolemia (Garcia *et al*, 2001).

In addition to these *bona fide* components of the machinery for vesicle formation (Table 1), mutations in the genes encoding PI phosphatases, for example enzymes that control organelle identity and membrane flux between compartments, have been found to cause human diseases. Most notable examples are the members of the myotubularin family of PI 3‐phosphatases, which comprise IDRs of variable lengths and regulate vesicular and nonvesicular membrane dynamics in the endolysosomal system and in autophagy (Table 1). Mutations in MTM1 (Laporte *et al*, 2000) and MTMR14 lead to inherited forms of centronuclear myopathy (Amoasii *et al*, 2012), while mutations in MTMR5, MTMR13 (Azzedine *et al*, 2003), and MTMR2 are associated with a specific subtype of Charcot Marie Tooth disease (i.e., CMT4B), a demyelinating sensory neuropathy. A closely related centronuclear myopathy similar to loss‐of‐function of MTM1 is due to dominant autosomal mutations in the endocytic IDP amphiphysin2/BIN1 (Lee *et al*, 2002; Nicot *et al*, 2007), possibly reflecting opposing roles of both proteins in the vesicular uptake and delivery of integrin molecules to the muscle surface. Finally, oculocerebrospinal syndrome of Lowe is a rare X‐linked disease characterized by congenital cataracts, glaucoma, intellectual disability, growth retardation, and renal tubule dysfunction caused by loss‐of‐function of the PI 5‐phosphatase OCRL (Hoopes Jr *et al*, 2005).

### Genetic disorders of IDPs involved in nonvesicular transport at MCS

One of the most crucial components of ER‐based MCS and, thereby, a critical regulator of nonvesicular lipid transport, are the ER membrane VAP‐A and VAP‐B proteins (Di Mattia *et al*, 2020). Mutations in VAP‐B (Mao *et al*, 2019; Borgese *et al*, 2021) have been linked to both ALS (see above) and spinal muscular atrophy, a rare neuromuscular disorder characterized by loss of spinal cord motoneurons (Table 2). These data suggest a close functional relationship between VAP‐B‐mediated nonvesicular lipid transport at ER‐based MCS and vesicular transport via AP5, SPG11/Spatacsin and ZFYVE26/Spastizin. The importance of nonvesicular transport at MCS is further underscored by the fact that mutations in the four human family members of the large yeast chorein‐domain lipid transport protein Vps13 (Leonzino *et al*, 2021; Melia & Reinisch, 2022) are all linked to disease. Vps13A‐D are structurally related proteins that act as lipid bridges between VAP proteins in the ER and various other organelles such as mitochondria (Vps13A; Subra *et al*, 2023), late endosomes/lysosomes (Vps13C; Kumar *et al*, 2018), and lipid droplets (Vps13A, Vps13C; Ramseyer *et al*, 2018; Chen *et al*, 2022), while Vps13B predominantly functions at the Golgi complex (Kolehmainen *et al*, 2003; Seifert *et al*, 2011). VPS13A loss‐of‐function pathogenic variants are characterized by a spectrum of movement disorders (chorea acanthocytosis, dystonia, tics, and sometimes parkinsonism; Rampoldi *et al*, 2001), Vps13B (also called COH1) mutations cause microcephaly (i.e., Cohen syndrome; Kolehmainen *et al*, 2003), and Vps13C is strongly implicated in Parkinson's disease (Lesage *et al*, 2016; Table 2). It is noteworthy that mutations in the IDR‐containing lipid phosphatases such as the PI 4−/5‐phosphatase Synaptojanin 1 (Krebs *et al*, 2013; Quadri *et al*, 2013) and the PI 4‐phosphatase Sac2/INPP5F (Cao *et al*, 2020) are also causally linked to Parkinsonism, implicating dysfunctional lipid metabolism in the disease.

**Table 2:** Examples of genetic disorders involving IDR‐containing proteins implicated in nonvesicular transport and MCS.

Gene	Protein function	Transport process	Known synaptic function	Disease	Ref.
VAP‐B	ER membrane protein forming MCS	Transport of diverse lipids across ER‐based MCS	No	Amyotrophic lateral sclerosis Spinal muscular atrophy	Borgese *et al* (2021), Mao *et al* (2019)
VSP13A	Lipid transport at ER‐based MCS	Transport of lipids at MCS between ER and mitochondria or lipid droplets	No	Chorea acanthocytosis	Rampoldi *et al* (2001)
VPS13B	Golgi scaffold, relationship to MCS is unclear	Unclear	No	Cohen syndrome (developmental disorder with microcephalie)	Kolehmainen *et al* (2003)
VPS13C	Lipid transport at ER‐based MCS	Transport of lipids at MCS between ER and late endosomes/lysosomes or lipid droplets	No	Parkinson's disease	Lesage *et al* (2016)
VPS13D	Lipid transport at ER‐based MCS	Transport of lipids at MCS between ER and mitochondria or peroxisomes	No	Spinocerebellar ataxia with spasticity and mitochondrial defects	Dziurdzik *et al* (2020)
SNCA	α‐Synuclein binds lipids	α‐Synuclein is implicated in SV reserve pool organization & possibly mitochondrial function via MCS	Yes	Parkinson's disease and related neurodegenerative disorders with Lewy bodies	Hoffmann *et al* (2021), Mukherjee *et al* (2023)
MAPT	Tau binds and stabilizes neuronal microtubules to facilitate axonal transport	Pathogenic Tau can bind SV membranes and thereby impair vesicle mobility and neurotransmission	Yes	Alzheimers's disease and other neurodegenerative disorders	Wegmann *et al* (2018), Zhou *et al* (2017)
UNC13A	Munc13‐1/UNC13A scaffold protein	Docks release‐ready SVs to the presynaptic active zone to enable fusion	Yes	Autism‐spectrum disorder (ASD)	Lipstein *et al* (2017)

### Genetic disorders of IDPs involved in membrane dynamics at synapses

Given the important roles of vesicular and nonvesicular membrane dynamics for neuronal function and the abundance of IDPs at synapses (Fig 5), it is not surprising that defects in IDP‐encoding genes with a role in vesicular and nonvesicular transport are implicated in neurodegeneration and neurological disease. Disease phenotypes in the brain may be further exacerbated by the fact that neurons are nondividing long‐lived cells and, thus, are particularly, vulnerable to defects in membrane homeostasis. A well‐known example of IDP‐driven synaptic dysfunction is the aggregation of α‐synuclein into the so‐called Lewy bodies in Parkinson's disease and Lewy body dementia (Table 2). α‐Synuclein is an IDP enriched within the presynaptic compartment that together with the SV‐associated IDP synapsin (Hoffmann *et al*, 2021; Mukherjee *et al*, 2023) regulates the partitioning of SVs between the phase‐separated vesicle reserve pool and the releasable pool of SVs (Milovanovic *et al*, 2018; Sansevrino *et al*, 2023). Synapsin dysfunction is also implicated in epilepsy (Garcia *et al*, 2004), a neuronal network disorder resulting from excitatory/inhibitory imbalance. A further example of a well‐known IDP is Tau, a microtubule‐binding protein implicated in more than 20 neurodegenerative diseases, including Alzheimer's disease by aggregating into phase‐separated β‐sheet amyloid‐like fibrils (Wegmann *et al*, 2018) that can spread in human brains. Elegant work in flies and rat neurons has shown that pathogenic Tau binds to synaptic vesicles via its N‐terminal domain and interferes with presynaptic functions, including synaptic vesicle mobility and release rate, resulting in impaired presynaptic neurotransmission (Zhou *et al*, 2017). This mechanism likely contributes to Tau pathology (Robbins *et al*, 2021). The endocytic protein PICALM, a clathrin‐associated IDP that controls the selective endocytosis of calcium‐permeable AMPA‐type glutamate receptors at postsynaptic sites, is implicated genetically in Alzheimer's disease (Harold *et al*, 2009; Jun *et al*, 2010; Table 1) and mutations in AP2, a clathrin adaptor involved in SV reformation and vesicular neurotransmitter endocytosis (López‐Hernández *et al*, 2022), are linked to epilepsy (Helbig *et al*, 2019). Strong genetic and functional links implicate the endocytic PI 4‐ and 5‐phosphatase synaptojanin (Miranda *et al*, 2018), an important regulator of vesicle uncoating at synapses, and the endosomal Retromer complex (Small *et al*, 2005) in Alzheimer's disease. Synaptojanin is a binding partner of the endocytic IDP Amphiphysin/BIN1, which has also been linked to Alzheimer's disease, and of Endophilin, a substrate of the LRRK2 protein kinase (McMahon & Boucrot, 2011). *LRKK2* is a major risk gene for Parkinson's disease (Deng *et al*, 2007; Floris *et al*, 2009). Mutations in the presynaptic IDP Unc13A (also called Munc13‐1), a key factor for regulated SV exocytosis, are causative of a variety of neurological diseases including autism‐spectrum and dyskinetic movement disorders (Lipstein *et al*, 2017). Finally, multiple lines of evidence link genes involved in autophagic vesicle formation to brain aging and neurodegeneration (Soreng *et al*, 2018; Bademosi *et al*, 2023).

These examples illustrate a critical role of IDPs involved in membrane dynamics at synapses for the function of the nervous system.

## Conclusions and perspectives

Given the complexity of membrane organization and dynamics, as well as the interplay between membrane‐bound compartments and IDPs recruited from or formed within the cytoplasm of eukaryotic cells and tissues, major knowledge gaps persist, in particular with respect to the nature of IDP‐organized assemblies *in vivo*. As conventional microscopy lacks the spatial and/or temporal resolution to capture the dynamics of IDP‐based protein assemblies, the size, composition, and dynamics of protein assemblies *in vivo* often remain unknown. Thus, in spite of the progress, major questions related to the function of IDR proteins in membrane dynamics remain unsolved (see Box 1: In need of answers).

Box 1 **In need of answers**

- What is the role and importance of specific molecular interactions (ordered assembly) vs. low‐affinity weakly adhesive condensate‐type interactions between IDR‐containing proteins (Musacchio, 2022) for vesicular and nonvesicular transport processes and subcellular membrane dynamics?
- Do IDPs and their condensates conceivably act as seeds in the assembly of large protein complexes such as vesicle coats or active zone scaffolds? If yes, how exactly do they operate?
- Do membranes modulate the biogenesis and dynamics of phase‐separated IDP condensates by serving as assembly platforms and/or by forming direct contacts?
- Do condensates represent a “storage form” of IDR‐containing membrane‐associated proteins that buffers their cytoplasmic pool by forming a specific chemical environment allowing proteins to maintain solubility akin to the postulated function of RNA granules?
- How are IDR‐promoted protein assemblies regulated by internal and external cues?
- How exactly do specific posttranslational modifications (i.e., IDR protein phosphorylation and acetylation) control IDP assemblies? How does their reversible association with ligands such as calcium or membrane phospholipids (e.g., PIs) control IDP assembly state?

So far, IDPs have been investigated with a broad range of methods, especially nuclear magnetic resonance (NMR) spectroscopy, small‐angle X‐ray scattering, electron paramagnetic resonance (EPR) spectroscopy, and single‐molecule fluorescence spectroscopy, often using integrative approaches in combination with molecular simulations or machine‐learning‐based approaches (see Chowdhury *et al*, 2023 and references within). An important next step will be to relate the properties of IDPs determined *in vitro* to their cellular functions. Ultimately, we seek to understand IDP interactions quantitatively within their native environment, for example, in cells and tissues (Plitzko *et al*, 2017). An important advantage of single‐molecule spectroscopy in this context is the ability to observe specifically labeled biomolecules even in complex environments (Follain *et al*, 2017). With the development of fast cameras and powerful localization algorithms for rapid and accurate localization of thousands of molecules, the nanometer localization and motion of hundreds and thousands of particles can be observed in live cells, even in the low‐millisecond regime, thereby enabling the visualization of IDP assemblies at nanoscale membrane sites and compartments.

Apart from having command over microscopic techniques allowing for following individual proteins (and lipids), and ensembles thereof, in complex, crowded environments, introducing appropriate labels into living cells or even the whole organism is greatly facilitated by the advent of highly efficient CRISPR/Cas9‐based gene editing. Particularly suitable preparations for *in vivo* single molecule imaging, as, for example, the *Drosophila* neuromuscular synapse, have recently allowed for an *in vivo* analysis of “plastic” state transitions of the IDP‐orchestrated active zone scaffold controlling the motility and density of voltage‐gated Ca^2+^ channels (Ghelani *et al*, 2023). We predict that similar approaches will be useful for other experimental models of vesicular and nonvesicular transport.

## Author contributions

**Volker Haucke:** Funding acquisition; visualization; writing – original draft; writing – review and editing. **Stephan J Sigrist:** Funding acquisition; visualization; writing – original draft; writing – review and editing.

## Disclosure and competing interests statement

The authors declare that they have no conflict of interest.
